# Low quality of routine microscopy for malaria at different levels of the health system in Dar es Salaam

**DOI:** 10.1186/1475-2875-10-332

**Published:** 2011-11-02

**Authors:** Judith Kahama-Maro, Valerie D'Acremont, Deo Mtasiwa, Blaise Genton, Christian Lengeler

**Affiliations:** 1 City Medical Office of Health, Dar es Salaam City Council, P.O. Box 63320, Dar es Salaam, United Republic of Tanzania; 2 Swiss Tropical and Public Health Institute, Department of epidemiology and Public Health, Socinstrasse 57, CH-4002 Basel, Switzerland; 3 Ministry of Health and Social Welfare, P.O. Box 9083, Dar es Salaam, United Republic of Tanzania; 4 University of Basel, Petersplatz, Basel, Switzerland

## Abstract

**Background:**

Laboratory capacity to confirm malaria cases in Tanzania is low and presumptive treatment of malaria is being practiced widely. In malaria endemic areas WHO now recommends systematic laboratory testing when suspecting malaria. Currently, the use of Rapid Diagnostic Tests (RDTs) is recommended for the diagnosis of malaria in lower level peripheral facilities, but not in health centres and hospitals. In this study, the following parameters were evaluated: (1) the quality of routine microscopy, and (2) the effects of RDT implementation on the positivity rate of malaria test results at three levels of the health system in Dar es Salaam, Tanzania.

**Methods:**

During a baseline cross-sectional survey, routine blood slides were randomly picked from 12 urban public health facilities in Dar es Salaam, Tanzania. Sensitivity and specificity of routine slides were assessed against expert microscopy. In March 2007, following training of health workers, RDTs were introduced in nine public health facilities (three hospitals, three health centres and three dispensaries) in a near-to-programmatic way, while three control health facilities continued using microscopy. The monthly malaria positivity rates (PR) recorded in health statistics registers were collected before (routine microscopy) and after (routine RDTs) the intervention in all facilities.

**Results:**

At baseline, 53% of blood slides were reported as positive by the routine laboratories, whereas only 2% were positive by expert microscopy. Sensitivity of routine microscopy was 71.4% and specificity was 47.3%. Positive and negative predictive values were 2.8% and 98.7%, respectively. Median parasitaemia was only three parasites per 200 white blood cells (WBC) by routine microscopy compared to 1226 parasites per 200 WBC by expert microscopy. Before RDT implementation, the mean test positivity rates using routine microscopy were 43% in hospitals, 62% in health centres and 58% in dispensaries. After RDT implementation, mean positivity rates using routine RDTs were 6%, 7% and 8%, respectively. The sensitivity and specificity of RDTs using expert microscopy as reference were 97.0% and 96.8%. The positivity rate of routine microscopy remained the same in the three control facilities: 71% before versus 72% after. Two cross-sectional health facility surveys confirmed that the parasite rate in febrile patients was low in Dar es Salaam during both the rainy season (13.6%) and the dry season (3.3%).

**Conclusions:**

The quality of routine microscopy was poor in all health facilities, regardless of their level. Over-diagnosis was massive, with many false positive results reported as very low parasitaemia (1 to 5 parasites per 200 WBC). RDTs should replace microscopy as first-line diagnostic tool for malaria in all settings, especially in hospitals where the potential for saving lives is greatest.

## Background

Malaria still poses a major threat to most countries in sub-Saharan Africa. Almost 40% of the global malaria episodes in 2008 were reported in only five African countries including Tanzania, and 85% of malaria deaths were in children under five years [1]. In Tanzania, malaria was until recently the leading cause of attendance in health facilities, with an estimated 16 million cases annually and 70,000 deaths [2]. Of all the malaria cases reported annually only 12-20% are confirmed parasitologically [3]. This is a common finding in the majority of African countries where the proportion of cases that have a confirmed diagnosis is usually less than 20% [1].

Prompt and accurate diagnosis is an essential component of malaria control strategies and enables the effective management of febrile patients [4]. For several years, it has been common practice to base diagnosis of malaria mainly on clinical signs and symptoms in health facilities across Africa due to the scarcity of laboratory facilities [5,6]. Similarly in Tanzania, many health facilities have insufficient laboratory facilities to support health services delivery. A recent survey conducted in Tanzania to assess health services provision in health facilities revealed that laboratory capacity to diagnose malaria was available in only 33% of health facilities, mostly in hospitals, and less in government than private health facilities [7].

Due to (1) the scarce availability of laboratory facilities and (2) the high mortality of malaria in young children, presumptive treatment for malaria in cases presenting with fever was seen as the only solution in the past and has been widely integrated in the daily practice of health workers [3,8,9]. Currently, there is a clear tendency to apply this strategy as well to children older than 5 years and even to adults, and this occurs usually regardless of malaria endemicity (i.e. also in settings with very low transmission of malaria) [10-12]. However, the shortcoming of this strategy lays in the difficulty to distinguish malaria from other febrile illnesses due to the overlap of clinical signs and symptoms and non-specific nature of these symptoms [13-15]. None of the symptoms and signs of malaria that were evaluated so far had sufficient sensitivity and specificity for the reliable diagnosis of a febrile patient [16,17]. As a result, the approach to diagnosing malaria on clinical grounds alone has led to substantial over-diagnosis [11,12,18,19]. The level of over-diagnosis is more pronounced in low transmission settings (including urban areas) where the proportion of fevers due to malaria is much lower [20,21].

Tanzania has recently changed its first line treatment for malaria through the introduction of artemether-lumefantrine (AL, Tradename Coartem™) [2]. Since AL is a more expensive drug than its predecessor sulphadoxine-pyrimethamine (SP), it is even more desirable to implement a reliable laboratory confirmation of malaria in the routine management of all fever patients. Firstly, this will prevent unnecessary treatments and the associated risk of adverse effects [15]. Secondly, this should reduce the potential development of drug resistance [22]. Thirdly, this will encourage health care providers to search for alternative causes of fever in malaria-negative patients, and particularly those that can become dangerous or even fatal if treatment is delayed [23-25]. Furthermore, fever illness episodes affect disproportionally people of low income, for whom the consequences of a misdiagnosis are the worst [26].

The current WHO recommendation has switched recently to the systematic testing of all fever cases [27], but this has been preceded by a vigorous debate [25,28]. Microscopy and rapid diagnostic tests for malaria (RDTs) are the methods currently recommended for parasitological confirmation of malaria and WHO recommends specifically the use of RDTs in lower level health units, where microscopy is usually not available. In public health facilities with laboratories the technique used for malaria diagnosis has traditionally been microscopy, commonly considered as the gold standard for testing malaria. In Tanzania, as in many other sub-Saharan countries, routine microscopy at primary level is known to be of low quality [12,29-31]. This is due to poor training, low skill level of laboratory staff, poor infrastructure, inadequacy of equipment and reagents, and lack of effective supervision [32]. Strangely, little is known about the quality of routine microscopy in higher level facilities, particularly hospitals or health centres. While the assumption is that quality of testing in these facilities is better than in dispensaries, there is little evidence to support this view.

On the other hand, RDTs have been used for routine malaria testing in a number of countries in Asia and Africa and have been shown to have high sensitivity and specificity [33-35]. Their advantage is their simplicity of use, the minimal training required and the ability to produce a result within a few minutes [36]. In Tanzania RDTs have also been used in recent years in a few research settings [30,37-40].

This study aimed at evaluating the quality of existing routine microscopy for malaria diagnosis in an urban setting in comparison to expert microscopy and RDTs, especially in hospitals and to assess the impact of RDT implementation on the malaria test result in health facilities in Dar es Salaam, United Republic of Tanzania.

## Methods

### Study area

Dar es Salaam is the economic capital of the United Republic of Tanzania, on the East coast of Africa. The current surface area of the city is 1,400 square kilometers and the population in 2002 was estimated to be about 2,500,000 [41]. With a growth rate of 4.3%, the population is presently estimated to be slightly above 3,000,000 [41].

Dar es Salaam has a hot and humid tropical climate with two rainy seasons, a main one observed during the months of March-May, and a short one occurring in November-December. Originally, it was an area with endemic and perennial malaria, with transmission occurring during the entire year. As a result of urbanization and malaria control activities it is now an area of low endemicity [42,43].

### Study setting

The study was conducted in the framework of the project 'Improving Malaria Diagnosis in Dar es Salaam health facilities' (IMALDIA) that was implemented in Dar es Salaam by the Dar es Salaam City Medical Office of Health in collaboration with the Swiss Tropical and Public Health Institute in Basel. IMALDIA aimed at assessing the impact of RDT implementation in the routine management of outpatients in this urban setting. It was conducted in 12 urban public health facilities with outpatient departments (OPD) in the three Municipalities of Dar es Salaam (Ilala, Kinondoni and Temeke). All three existing municipal hospitals (corresponding to district hospitals) were included, and the other nine health facilities were selected among the13 eligible centres/dispensaries in collaboration with Council Health Management Teams (CHMT) from the Municipal Medical Offices of Health. Selection was done first according to the number of patients seen per day (at least 100 consultations per day) and the willingness of health facility staff to participate, and then to the criteria for matching described below.

### Study design and interventions

All three hospitals were included as intervention health facilities and no controls were available for them. For the nine health centres/dispensaries, we had one trio of facilities in each of the three Municipalities that were matched based on availability of microscopy, accuracy of the general registers (called MTUHA books) and laboratory registers, socio-economic status of the population served and quality of governance of the health facility. Two facilities per trio were then randomly assigned as intervention facilities and one as control health facility. The control facilities had no training and no RDT introduced.

In February 2007, a one-day sensitization meeting was conducted with health facility managers and representatives from the CHMTs of each district to build awareness on the role of RDT as a tool for diagnosis of malaria, and to discuss the logistics of RDT implementation. The intervention was carried out in a way as close as possible to programme conditions and consisted of the following: (1) training of 181 health workers divided in five identical sessions of one day each, (2) introduction of RDT at the end of March 2007, and (3) on-site supervision after 1, 2, 5, 10 and 15 months. Besides background information on RDTs, the training focused on how to select cases for RDT testing, how to use the test result and discussions around real-life case studies. As part of the training there was also a demonstration on how to perform the RDT while referring to the job aid. Each participant was given the opportunity to perform the test on a fellow participant and to interpret the result. The key take-home messages were: (1) to perform an RDT only in patients with a history of fever or elevated temperature, and (2) not to give anti-malarials to patients with a negative test. The participants were also instructed to investigate other causes of fever using the IMCI tool, especially in the event of a negative RDT. The RDTs were introduced in April 2007.

### Data collection

*Data Source 1*. During a baseline cross-sectional survey of health facilities conducted from November 2006 to January 2007, a total of 346 blood slides performed routinely at health facility laboratories were picked randomly from each of the 12 selected health facilities for three consecutive days. At the health facility thick blood smears were prepared and stained with 10% Giemsa stain. Microscopy reading was done to establish presence of malaria parasites and their density. These blood slides were then sent for blind expert microscopy reading at the Muhimbili Consultant Teaching and Referral Hospital reference laboratory. Expert reading was performed by a principal laboratory technologist and scientist with 30 years of experience in his field. The minimum reading time spent per slide was 7 minutes and parasite density was determined by counting the number of asexual parasites per 200 white blood cells. A smear was declared negative by the expert if no parasites were seen after examining 100 high power fields. Double reading was not done. Sensitivity and specificity, positive and negative predictive values were calculated using expert microscopy as gold standard.

*Data Source 2*. Routine health service statistics were collected from health facility register books used in all the 12 health facilities and known as MTUHA (abbreviation in Kiswahili language meaning "Health Management Information System"). The aim was to get the total number of laboratory tests for malaria performed every month, and the number of positive test results for routine microscopy and RDTs. These data were collected from January 2006 to September 2008.

*Data Source 3*. During the months of May and June 2007 (rainy season) and the months of October and November 2007 (dry season), two cross-sectional surveys were conducted in Buguruni Health Centre, an urban public health facility in Ilala Municipality, in the centre of the city. The aim was to determine the malaria positivity rate in febrile patients, based on RDTs. The inclusion criteria were: (1) patients coming for a first consultation (not follow-up visit), (2) patients with a history of fever in the last 48 hours (or with a measured auxiliary temperature ≥ 37.5°C), and (3) being resident of the catchment area of the health facility. Exclusion criteria were: (1) signs of severe illness needing referral, and (2) if the main complaint of the patient was an injury or trauma. Following a clinical consultation, an RDT for malaria was performed for each patient. During the rainy season, a blood slide for expert microscopy reading was performed in parallel to the RDT.

### Data analysis and ethical clearance

To calculate the positivity rate of routine microscopy or RDT reported in the MTUHA books, the average of the total number of tests performed monthly and the average of positive tests for each health facility were first calculated. To calculate the mean positivity rate for all three hospitals the sum of the monthly average of positive tests in all three hospitals was divided by the sum of the monthly average of the total number of tests performed. The same methodology was used to calculate the weighted monthly average health centres and dispensaries. All data were entered and verified in Microsoft Excel (Microsoft Corp., Seattle, USA) and EpiInfo 2000 for Windows version 3.5.1 and checked for errors by referring to the original data collection files. All analysis were performed using STATA version 10.

This work was carried out in compliance with the principles of the Helsinki Declaration. Ethical approval for the study was obtained from the National Institute of Medical Research (NIMR) in Tanzania (Reference Number NIMR/HQ/R.8a/Vol.IX/418). Permission to conduct the study in the health facilities was granted by the Dar es Salaam Regional and Municipal health authorities. Informed consent was obtained from the patients and caretakers for the health facility surveys.

## Results

### Test performance of routine microscopy at baseline

From November 2006 to January 2007, a total of 346 random blood slides performed routinely in 11 health facilities were taken from the laboratory after the slide had been examined by the usual microscopist. In one health facility (dispensary 1) no staff was available for slide reading during the days of the survey. Of these 346 slides, results of expert microscopy were missing for two slides and the routine results for nine slides could not be obtained because the patients did not return to the clinicians to complete the consultation process. Of the remaining 335 slides, 178 (53.1%) slides were reported positive by health facility routine microscopy but only 7 (2.1%) were positive by expert microscopy. The positivity rates of malaria by routine and expert microscopy for each health facility are summarized in Table 1. As can be seen, despite the small numbers of slides examined per health facility the positivity rate differs consistently between routine and expert microscopy, with the exception of hospital 2 which had a much lower positivity rate by routine microscopy.

**Table 1:** Positivity rate of malaria by routine and expert microscopy in a cross-sectional survey of 12 health facilities at baseline (before RDT implementation), Dar es Salaam (n = 335).

Name of Health facility	No. of slides examined	No. positive routine	% positive routine	No. positive expert	% positive expert
Hospital 1	22	9	40.9%	1	4.5%
Hospital 2	15	2	13.3%	0	0.0%
Hospital 3	33	21	63.6%	1	3.0%
Health Centre 1	40	35	87.5%	0	0.0%
Health Centre 2	30	8	26.7%	3	10.0%
Health Centre 3	30	10	33.3%	2	6.7%
Dispensary 1	0	N.A	N.A	N.A	N.A
Dispensary 2	29	19	65.5%	0	0.0%
Dispensary 3	36	22	61.1%	0	0.0%
Control 1	32	8	25.0%	0	0.0%
Control 2	18	10	55.6%	0	0.0%
Control 3	50	34	68.0%	0	0.0%

**Total**	**335**	**178**	**53.1%**	**7**	**2.1%**

Dispensary 1 did not perform any microscopy during the survey. N.A = not applicable.

Using expert microscopy with a single reading as a comparator, routine microscopy had a sensitivity of 71.4% [CI: 35.9%, 91.8%] and a specificity of 47.3% [CI: 41.9%, 52.7%]. The positive and negative predictive values were 2.8% [CI: 1.2%, 6.4%] and 98.7% [CI: 95.5%, 99.6%], respectively. Of the 178 slides that were positive by routine microscopy, only two slides were reported as having a parasitaemia level above 10 per 200 white blood cells (WBC)-these were from the same health centre and were both positive by expert microscopy (parasitemia of 1160 and > 9999 parasites per 200 WBC)-and 165 (92.7%) were reported as having very low densities of one to five parasites per 200 WBC. Of the 157 slides that were negative by routine microscopy, two slides were positive when re-read by the expert with both high parasitaemia (1226 and 2120 parasites/200 WBC). Five additional slides were positive by expert microscopy with parasite densities of 96, 406, 1160, > 9999 and > 9999 per 200 WBC, while the routine microscopy at HF gave corresponding parasite densities of 2, 5, 50, 10 and 100. There was thus a marked difference between the parasite densities reported by routine microscopy and those reported by expert microscopy. The median parasite density for routine microscopy was only three parasites per 200 WBC, whereas the median density for expert microscopy was 1226 parasites per 200 WBC. Clearly, laboratory technicians often generated false positives by reporting a very low density. If a cut-off parasite density of more than five per 200 WBC had been used for the results of routine microscopy, the specificity would have increase to 97.0% but at the cost of a reduced sensitivity of 42.9%. This would, however, not have affected the NPV which would have remained unchanged (98.8%).

### Effect of RDT implementation on malaria test positivity rate reported in the MTUHA books

From April to December 2006, the average number of blood slides performed each month in the 9 intervention health facilities was 20,386. The mean positivity rates (PR) of the routine microscopy were 43% in hospitals, 62% in health centres and 58% in dispensaries (range per facility: 14 to 93%). In the following year (post RDT initiation) and during the same period from April 2007 to December 2007 (to account for the influence of seasonality), an average number of 25,922 RDTs and 1191 blood slides were performed per month. The mean PR of routine RDTs in hospitals, health centres and dispensaries were 6%, 7% and 8% respectively (range per facility: 5 to 12%). By contrast, over the two-year period the mean PR of routine microscopy remained the same in the three control health facilities: 71% before and 72% after RDT introduction. Table 2 shows the average number of malaria tests performed per month in each health facility, both for microscopy (period before RDT introduction) and for RDTs (after RDT introduction), the number of positive tests and the positivity rate with confidence intervals. Again there is a consistent and marked difference between the two testing methods, with the exception of Buguruni Health Center which appeared to be performing better for microscopy.

**Table 2:** Malaria test positivity rates based on health statistics registries using routine microscopy (from April to December 2006, before the intervention) and routine RDT (from April to December 2007, after RDT implementation) in the 12 selected health facilities in Dar es Salaam.

	Routine Microscopy			Routine RDTs		
Health Facility	Monthly average total tests	Monthly average positive tests	Positivity rate% [95% CI]	Monthly average total tests	Monthly average positive tests	Positivity rate% [95% CI]
**Hospitals**						
Mwananyamala	6505	2548	39.2 [38.0, 40.4]	5412	316	5.8 [5.2, 6.5]
Amana	1846	592	32.1 [30.0, 34.2]	3595	202	5.6 [4.9, 6.4]
Temeke	4761	2499	52.5 [51.1, 53.9]	5949	307	5.2 [4.6, 5.8]
**Health Centres**						
Tandale	3213	2980	92.7 [91.8, 93.6]	3823	230	6.0 [5.3, 6.8]
Buguruni	1857	260	14.0 [12.5, 15.7]	2531	186	7.3 [6.4, 8.4]
Kigamboni	655	321	49.0 [45.2, 52.8]	1060	69	6.5 [5.2, 8.2]
**Dispensaries**						
Kawe	307	213	69.4 [64.0, 74.3]	561	35	6.2 [4.5, 8.6]
Tabata A	1005	479	47.7 [44.6, 50.8]	1670	76	4.6 [3.7, 5.7]
Mbagala K	238	202	84.9 [79.8, 88.9]	1319	158	12.0 [10.3, 13.8]
**Control facilities**						
Sinza	987	733	74.3 [71.4, 76.9]	1908	1620	84.9 [83.2, 86.4]
Vingunguti	723	466	64.5 [60.9, 67.9]	1411	600	42.5 [40.0, 45.1]
Mbagala R	469	342	72.9 [68.7, 76.7]	549	546	99.5 [98.5, 99.8]

**Figure 1:**
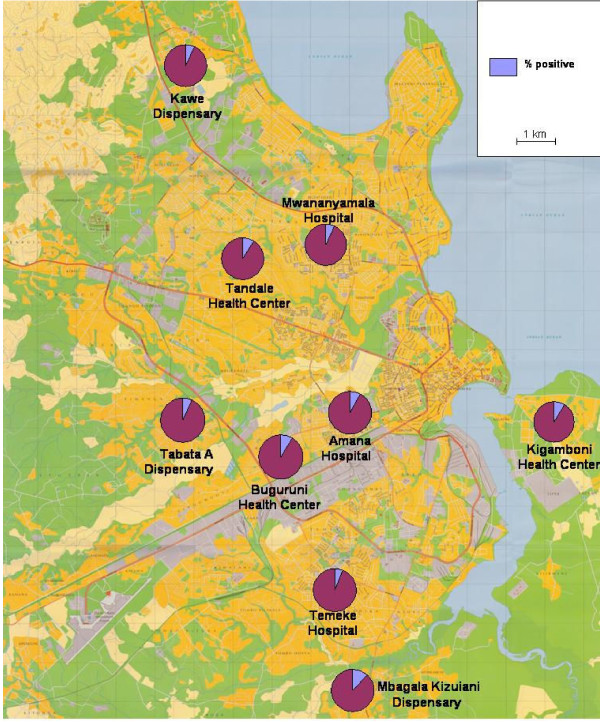
**Geographic distribution of health facilities and malaria positivity rates by RDT in patients in Dar es Salaam (exact values in text)**.

**Figure 2:**
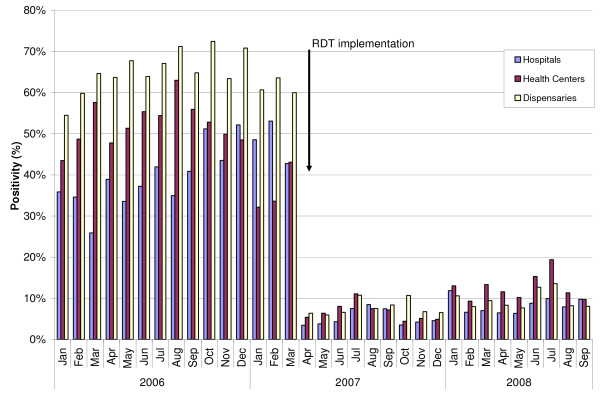
**Malaria test positivity rates in intervention health facilities, before and after RDT implementation in April 2007**.

**Figure 3:**
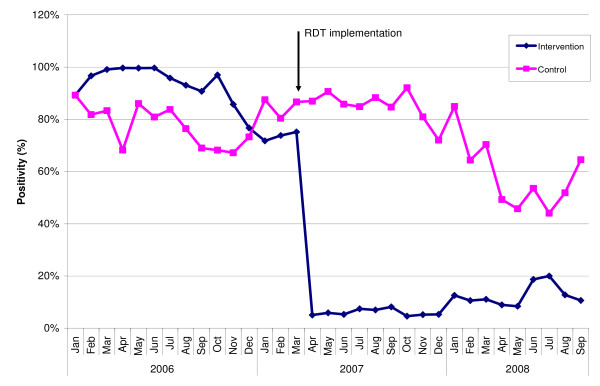
**Malaria test positivity rates in one intervention and one matched control health centre before and after RDT introduction (see text for details)**.

When considering the whole post-intervention period, from April 2007 to September 2008, the mean test positivity rates using RDTs were 7% at hospitals, 9% at health centres and 9% at dispensaries (range 6 to 12%) (Figure 1). Figure 2 shows the malaria test positivity rates over time-by routine microscopy up to March 2007 and by routine RDT from April 2007 onwards-in the nine intervention facilities. There is an instantaneous and marked reduction in positivity rates following the introduction of RDTs. Figure 3 shows the malaria test positivity rates in one intervention (Mbagala Kizuiani) and in one matched control health centre (Mbagala Rangi tatu) during the whole period of the study.

### Malaria positivity rates in febrile patients in the rainy and dry seasons in Buguruni Health Centre

In May-June 2007 (rainy season), 602 febrile patients were included in the Buguruni Health Centre sub-study, of which 337 (56.0%) were children under 5 years of age. The overall number of fever patients who tested positive for malaria by RDT was 82 (13.6%). The rates were 13.1% in children under 5 years and 14.3% in patients above five years. Using expert microscopy from the Muhimbili University laboratory as reference, the sensitivity of the RDTs was 97.0% and their specificity was 96.8%. The positive and negative predictive values were 79.2% and 99.6%. In a similar survey in October-November 2007 (dry season), 333 out of 602 (55.3%) of the patients recruited were under five years of age. The number of patients with fever who tested positive for malaria by RDT was just 20 (3.3%).

## Discussion

The results of this study showed that there was poor performance of routine microscopy in health facilities at all levels of care. Although hospital two, with a positivity rate of only 13.3% by routine microscopy, seemed to perform better in terms of specificity, it is difficult to comment on individual health facilities due to the small numbers of slides collected. However, as group the hospitals did not seem to perform better than the health centres or dispensaries, despite being considered as referral centres providing more specialized and higher quality services [44]. The low sensitivity (although not measured precisely due to the very small number of true malaria cases) and specificity of routine microscopy we observed in this study are consistent with earlier studies done in Tanzania [11,38,45] and elsewhere in Africa across different malaria transmission areas [12,29,46]. This poor performance has profound clinical implications for two main reasons. Firstly, the low sensitivity leads to malaria cases being missed and therefore patients being denied a safe treatment for a potentially lethal disease. Fortunately, the prevalence of malaria in Dar es Salaam is very low, hence this was less an issue in this setting. On the other hand, the low specificity with an associated very low positive predictive value of 2.8% leads to a massive over-diagnosis and subsequent over-treatment with antimalarials, a practice commonly experienced in low to moderate transmission settings in Africa [21,29,47]. The low specificity of routine microscopy has many negative consequences such as a waste of limited resources, increased costs for drugs, unnecessary exposure of patients to the adverse effects of drugs, and chiefly the fact that clinicians tend to overlook other causes of febrile illness [24,25]. Finally, an analysis of these data will give a completely incorrect picture of the epidemiology of malaria, with serious implications for the management of patients and the planning of control measures [27].

Further evidence for the reporting of many false positive results by the microscopists was the fact that 93% of all positive findings were reported as very low parasitaemia (1-5 malaria parasites per 200 white blood cells), which contrasted with the results of expert microscopists reporting a median parasitaemia of more than 1000 malaria parasites per 200 WBCs. Hence, it is very likely that because they knew the poor quality of their microscope, slide preparation and staining (and thus the difficulty in differentiating real parasites from artifacts), the microscopists simply wrote down having seen a few parasites to avoid taking any risk.

In the present study we observed a reduction of more than 80% in the proportion of malaria positive tests in the intervention facilities following the introduction and routine implementation of RDTs. This reduction was observed in all health facility levels and type of facility, i.e. hospitals, health centres and dispensaries. The effect was so marked and coincided so well with the introduction of RDTs that it is almost certain to be causally related to the improved diagnostic performance of the malaria test. This conclusion is reinforced by the fact that the RDT results were confirmed by expert microscopy. As a result of this reduction, the consumption of AL decreased by 68% [47], and this approach could be shown to be safe for the patients [48].

The poor performance of routine microscopy may be attributed to several factors, including lack of skilled and competent laboratory technicians, and also an inadequate number of laboratory personnel, who are overwhelmed by the large volume of blood slides requested by clinicians. At the hospitals in Dar es Salaam, the number of slides performed per technician ranges from 100 to 200 per day, while at health center level it ranges from 70 to 100 per technician per day. Other factors that contributed to this situation are the poor condition of laboratory equipment (especially microscopes), insufficient or substandard reagents, frequent power interruptions, ambiguous guidelines, inadequate supervision and virtually non-existent quality control systems [6,30,32,34,37,49]. An in-depth assessment of these factors was not within the scope of our study.

In some places in Africa, efforts to improve microscopy have been successful, such as in Ghana [50] and Uganda [51]. However, it is uncertain how such isolated efforts can be sustained in the long-term when implemented routinely and on a large scale [31,34]. This is illustrated by a study in a rural district of Tanzania which documented that there had been no substantial improvement in the performance of microscopy despite intensive refresher training for laboratory technicians and supervision [45]. Training needs to be properly planned, regular and considering the high turn-over of health staff. Moreover, other important structural factors need to be addressed, such as levels of laboratory staffing, ensuring adequate supportive supervision, provision and maintenance of essential laboratory supplies and improving the basic health facility infrastructure. In view of this, achieving substantial improvements in malaria diagnosis by microscopy requires an enormous investment that has to be weighted against the benefits of introducing RDTs. Finally, the recognized suboptimal quality of microscopy in health facilities appears to have led clinicians throughout Africa to mistrust the results, and to prescribe anti-malarials to patients regardless of the test result [2,6,11,12,29,32].

The large reduction in the malaria positive rates seen in the intervention facilities after RDT implementation were not observed in the control facilities, despite a slight decrease in the positivity rates observed during the second half of RDT implementation. This late trend may have been due to progressive 'contamination' of the control facilities due to transfers of staff from intervention to control health facilities or to informal inter-facility communication (no formal training on microscopy took place during the study period).

The true prevalence rate of malaria in feverish patients in our study was found to be below 10% in all health facilities. The facilities were fairly well distributed across the three municipalities of Dar es Salaam and since they served a significant proportion of the population we can consider our results to be representative for Dar es Salaam. This clearly indicates that the prevalence of malaria in Dar es Salaam is much lower than has been previously documented [3]. The low malaria positivity rates are consistent with results from an earlier study done by Wang *et al *in Dar es Salaam during the dry season, which found less than 7% of all fever to be due to malaria [43]. These rates also compare well with a recent Malaria Indicator Survey which found a prevalence rate in children less than five years in Dar es Salaam Region of 1.2% [52].

### Study limitations

This assessment was based on data collected from routine health service statistics recorded in health facility register books (MTUHA), which may have been inaccurate or incomplete. When in doubt, the data from other health facility sources (laboratory registries) were counterchecked for consistency. In any case, the considerable reduction in the proportion of positive tests for malaria following the introduction of RDTs is very unlikely to have been due simply to erroneous records.

The implementation of the study focused on public health facilities, so these findings may not be generalized to private health facilities, which see the smaller part of health consultations in Dar es Salaam [53]. Anecdotal evidence suggests that the malaria positivity rates for malaria microscopy are even higher in private health facilities, but we could not investigate this in the present study. This is certainly a priority for future research.

## Conclusions

One of the key strategies of the Tanzania National Malaria Control Programme is to increase the proportion of confirmed malaria cases from the current level of 20% to 50% by 2012 [2]. This might be difficult to achieve with microscopy, considering the numerous constraints that appear to compromise its quality. RDTs therefore offer an exceptional opportunity for providing rapid and accurate diagnosis, particularly in outpatient settings where it is critical to exclude safely and rapidly malaria to enable timely decision-making and rational prescribing practices [54,55]. It should be noted, however, that in order to maximize the benefits of RDTs it is important that their deployment is preceded by comprehensive training of health care providers tailored to their daily clinical practice [47,54,56]. Training of this kind will enable clinicians to request RDT testing for appropriate indications, to interpret results correctly and take them into account when prescribing treatment. With the rapidly decreasing malaria prevalence in many endemic settings, additional efforts need to be made urgently to develop capacities for health facilities and clinicians to confirm and manage differential diagnosis of non-malarial febrile conditions.

## Competing interests

The authors declare that they have no competing interests.

## Authors' contributions

JKM and VDA designed and organized the study in the field, were responsible for overall supervision of study implementation and analyzed the data. JKM wrote the initial manuscript and VDA and CL revised it. DM, BG and CL contributed to conception and design of the study and DM and BG reviewed the manuscript. All authors read and approved the final manuscript.
